# Clinical and Epidemiological Characteristics of Postdischarge Patients With COVID-19 in Tehran, Iran: Protocol for a Prospective Cohort Study (Tele-COVID-19 Study)

**DOI:** 10.2196/23316

**Published:** 2021-02-02

**Authors:** Laya Jalilian Khave, Mohammad Vahidi, Dorsa Shirini, Ghazal Sanadgol, Farzad Ashrafi, Mehran Arab-Ahmadi, Alireza Fatemi, Minoosh Shabani Barzegar, Taha Hassanzadeh, Behandokht Rezaei, Alireza Zali, Davood Ommi, Shabnam Nohesara, Reza Jalili Khoshnood, Saeed Abdi, Ali Pirsalehi, Ehsan Masarat, Mostafa Shokoohi, Mohammad Karamouzian

**Affiliations:** 1 Faculty of Medicine Shahid Beheshti University of Medical Sciences Tehran Iran; 2 Functional Neurosurgery Research Center Shohada Tajrish Neurosurgical Center of Excellence Shahid Beheshti University of Medical Sciences Tehran Iran; 3 Advanced Diagnostic and Interventional Radiology Research Center Tehran University of Medical Sciences Tehran Iran; 4 Taleghani Hospital Research Development Committee Shahid Beheshti University of Medical Sciences Tehran Iran; 5 Infectious Diseases and Tropical Medicine Research Center Shahid Beheshti University of Medical Sciences Tehran Iran; 6 Mental Health Research Center Psychosocial Health Research Institute Iran University of Medical Sciences Tehran Iran; 7 Kashan University of Medical Sciences and Health Services Kashan Iran; 8 Dalla Lana School of Public Health University of Toronto Toronto, ON Canada; 9 HIV/Sexually Transmitted Infection Surveillance Research Center, and World Health Organization Collaborating Center for HIV Surveillance Institute for Futures Studies in Health Kerman University of Medical Sciences Kerman Iran; 10 Faculty of Medicine School of Population and Public Health University of British Columbia Vancouver, BC Canada

**Keywords:** cohort studies, COVID-19, health care delivery, Iran, medical education, telemedicine

## Abstract

**Background:**

COVID-19 was declared a pandemic on March 11, 2020. Given that the severe shortage of hospital beds has led to early discharge and insufficient patient education on home care routines and isolation protocols, the close follow-up of patients and their immediate relatives is an integral part of transitioning from hospital care to home care for patients with COVID-19.

**Objective:**

We designed the Tele-COVID-19 prospective cohort to follow-up with COVID-19 patients in Tehran, Iran, and improve health care delivery and the recording of postdischarge patients’ clinical profiles.

**Methods:**

All adult patients who were admitted to the COVID-19 wards of teaching hospitals in Tehran, Iran were eligible to participate in this cohort study. At baseline, patients were recruited from 4 major hospitals from March 9, 2020 to May 20, 2020. Telephone follow-ups, which were led by volunteer medical students, were conducted on postdischarge days 1-3, 5, 7, 10, and 14. We collected data on a range of sociodemographic, epidemiological, and clinical characteristics by using a standard questionnaire.

**Results:**

Of the 950 patients with confirmed COVID-19 who were approached, 823 (response rate: 86.6%) consented and were enrolled into the cohort. Of the 823 participants, 449 (54.5%) were male. The mean age of participants was 50.1 years (SD 12.6 years). During the initial data collection phase, more than 5000 phone calls were made and over 577 reports of critical patients who were in need of urgent medical attention were recorded.

**Conclusions:**

The Tele-COVID-19 cohort will provide patients with sufficient education on home care and isolation, and medical advice on care and the proper use of drugs. In addition, by preventing unnecessary hospital returns and providing information on household SARS-CoV-2 transmission as early as possible, this cohort will help with effective disease management in resource-limited settings.

**International Registered Report Identifier (IRRID):**

DERR1-10.2196/23316

## Introduction

The COVID-19 disease, which is caused by the SARS-CoV-2 virus, was declared a pandemic on March 11, 2020. Based on the existing evidence, the risk of infection appears to be relatively low for the general population. However, older people, immunocompromised people, and those with underlying health conditions, such as cardiovascular diseases, are at an elevated risk of morbidity and mortality [1]. As of December 11, 2020, 71,088,688 patients with confirmed COVID-19 and 1,595,096 deaths have been reported across the globe [2]. To date, effective treatment options for COVID-19 are unavailable. However, more than 2500 trials and studies are being conducted worldwide to develop and evaluate different therapeutic options for COVID-19 [3]. Implementing swift, community-centered preventive measures, providing timely diagnoses, treatments, contact tracing services, and methods for the successful isolation of patients at home, and reducing the household transmission of the virus among infected patients’ close contacts have been at the core of recommended strategies for combating the disease [4-6].

Despite the extensive worldwide efforts for controlling the pandemic, the constantly rising patient load and limited personal protective equipment supplies have overwhelmed health care systems across the globe. However, the toll of the COVID-19 pandemic has been heavier for low- and middle-income settings, wherein health care systems are already underfunded, understaffed, and overstretched. Iran is one of the countries that was hit the hardest by the COVID-19 outbreak. The first patient with confirmed COVID-19 in Iran was reported on February 19, 2020 in the city of Qom, which is 200 km away from Tehran, the capital city of Iran. As of December 11, 2020, 1,092,407 patients with confirmed COVID-19 and 51,727 deaths have been reported in the country [7]. Iran’s initial response to the pandemic included physical distancing control policies that aimed to minimize close contact within communities, as well as individual-level restrictions (eg, quarantine and isolation) and community-level restrictions (eg, educational and recreational facility closures, nonessential business closures, and the cancellation of public/mass/crowded gatherings). Nevertheless, economic sanctions, inadequate financial and human resources, inefficient leadership, and limited hospital capacities for the rapidly growing number of patients with COVID-19 who require hospitalization have created real and considerable challenges for controlling the epidemic [8-10]. As >98% of the population has access to mobile or landline phones [11], a cost-effective method for reducing the community transmission of SARS-CoV-2 and managing the influx of patients with COVID-19 in Iran's hospitals could be managing discharged patients or patients with noncritical conditions via routine telephone follow-ups. Telemedicine has been proposed as an effective approach for responding to health emergencies. A telemedicine approach may ensure that the limited number of hospital beds are occupied by the people who need them most, and provide patients and their families with access to medical care, without unnecessary referrals to health care facilities [12,13]. Therefore, we designed a prospective cohort study (ie, the Tele-COVID-19 study) to follow-up with patients with COVID-19 who have been discharged from teaching hospitals in Tehran, to assess the overall spread of SARS-CoV-2 in the community. The specific objectives of the Tele-COVID-19 study are as follows: (1) conduct telephone-based follow-ups with patients who were admitted to the coronavirus emergency departments of certain hospitals in Tehran; (2) precisely monitor postdischarge patients’ signs and clinical symptoms; (3) provide patients with sufficient education on home care and isolation principles, and medical advice on care and the proper use of drugs; (4) prevent unnecessary hospital returns and provide timely information on the in-household transmission of SARS-CoV-2; and (5) refer postdischarge patients with critical conditions to the emergency department in a timely manner, and facilitate patient readmission when necessary. Herein, we present the overall characteristics of the Tele-COVID-19 cohort and the preliminary baseline characteristics of the first-round patients who were recruited into the cohort.

## Methods

### Study Design

The Tele-COVID-19 study is a prospective cohort study that was designed to follow-up with postdischarge patients with COVID-19 for a 2-week period in Tehran, Iran. At baseline, data collection was completed by a medical student volunteer group, which was established on February 22, 2020 under the supervision of clinical professors [14]. Baseline data collection was conducted from March 9, 2020 to May 20, 2020. Given the prospective nature of the cohort, a top-up sample of participants will be included in the sampling frame during subsequent waves of data collection. Due to the emerging nature of SARS-CoV-2, a prospective sample size was not calculated. Additionally, because the recruitment of patients depends on the spread of COVID-19 in Tehran, there is no specific end date for recruitment at this point in time.

### Setting

**Figure 1:**
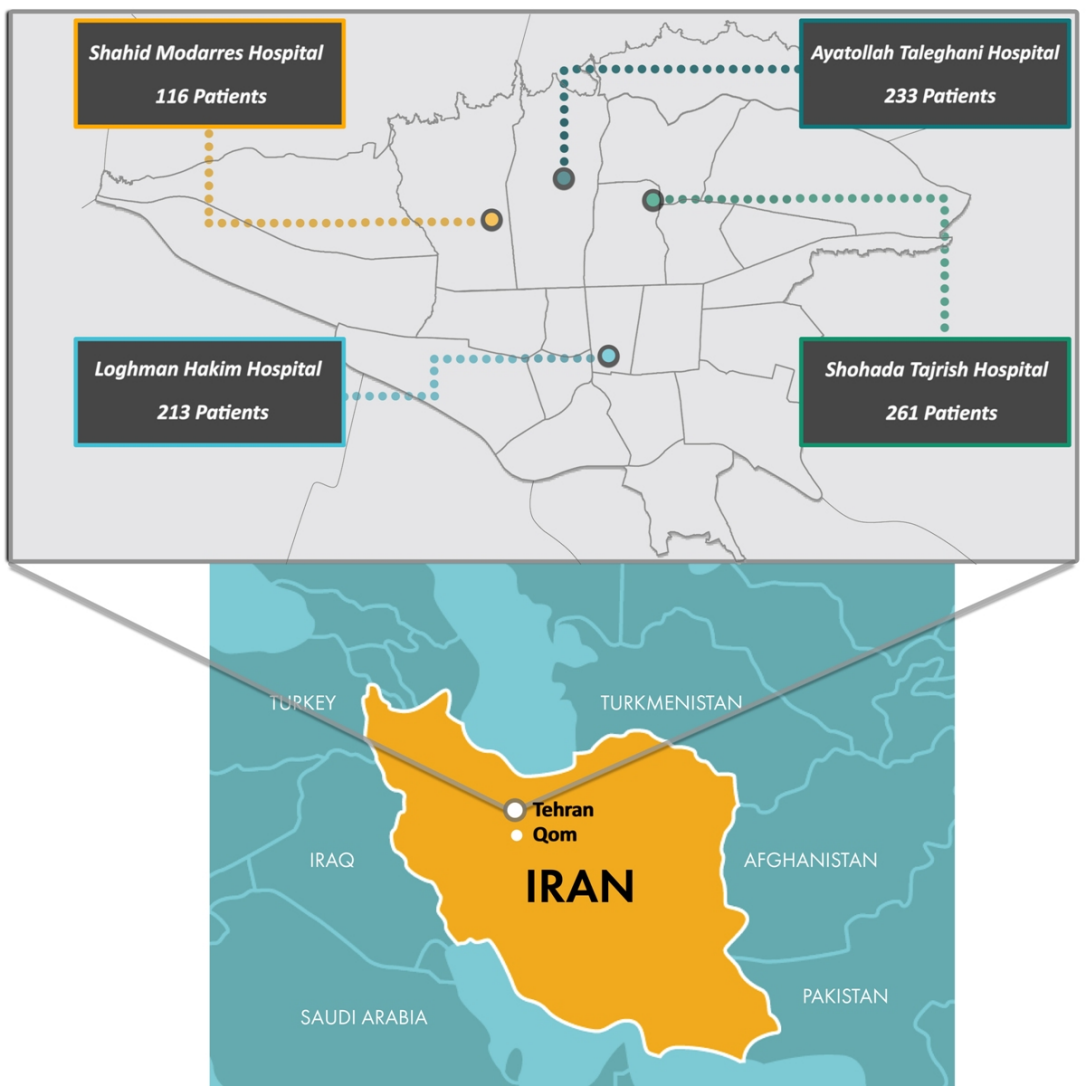
Hospitals included in the first phase of recruitment for the Tele-COVID-19 cohort.

In total, 4 major teaching hospitals in Tehran (ie, Shohada Tajrish Hospital, Ayatollah Taleghani Hospital, Shahid Modarres Hospital, and Loghman Hakim Hospital) were included at baseline. Collectively, these hospitals have 1652 beds, 136 primary care clinics, more than 100 specialties, and over 3000 employees. Of these 4 hospitals, 3 are located in the northern districts of Tehran and 1 is located in the southern district (Figure 1).

### Eligibility Criteria

Eligible participants were hospitalized adults (ie, aged >18 years) with COVID-19. Patients who tested positive for SARS-CoV-2 infection in polymerase chain reaction tests, and those who were treated and discharged from COVID-19 wards were recruited into the cohort. Participants were excluded if their contact information was wrong or missing. All patients who did not respond to follow-up phone calls or declined to provide consent were also excluded. Participants were briefed about this study’s aims and objectives via in-person discussions in the hospital and postdischarge phone calls. All participants provided verbal consent, and each patient received a unique national identification code to avoid potential biases that could arise from patients who went to several other health care facilities postdischarge.

#### Data Collection

At baseline, telephone follow-ups, which were led by volunteer medical students, were conducted on postdischarge days 1-3, 5, 7, 10, and 14, in accordance with a predetermined protocol and under the supervision of clinical professors. The calls were made to patients’ cell phones or landline phones from 10 AM to 4 PM. In the case of a nonresponse, calls were repeated 3 times at 2-hour intervals until the end of the calling time. Patients were excluded if they could not be reached by the end of the day. The first round of interviews was conducted by medical clerks. If a patient was assessed to have a concerning or critical condition, a follow-up phone call was made by a senior medical intern after consulting clinical professors for further assessment. Data were recorded on a secure and password-protected web-based platform. Each medical clerk signed a nondisclosure agreement before the enrollment period, to ensure that data remained secure and confidential. Medical clerks could only observe the data that they themselves collected; they could not observe the data that other team members collected. By the end of each patient’s 14-day follow-up period, all data were extracted from the web-based platform and stored on a password-protected external hard drive.

Data were collected by using a pilot-tested, comprehensive COVID-19 risk assessment questionnaire. The development of the questionnaire was informed by the Center for Disease Control and Prevention and the national guidelines of the Ministry of Health and Medical Education, and included the following areas: sociodemographic information (eg, age and sex), a history of potential exposure to SARS-CoV-2 (eg, a travel history to China or the city of Qom and a history of exposure to patients with COVID-19), signs and clinical symptoms (eg, fever, dry cough, dyspnea, nausea, and diarrhea), a medical history of underlying conditions (eg, a history of diabetes, cardiovascular disorders, chronic lung disease, chronic renal disease, and immunodeficiency), habitual history (eg, current smoker, former smoker, and nonsmoker), prescribed drugs at postdischarge (eg, hydroxychloroquine and lopinavir/ritonavir), a history of non-COVID-19–related medications (eg, nonsteroidal anti-inflammatory drugs and statin), and household information on close contacts (eg, high-risk household contacts and household transmissions).

#### Interview Process

Interviewers were enrolled if they were fourth-year to seventh-year medical students and registered in their respective medical schools at the time of interviews. There were 2 main groups of interviewers, as follows: (1) medical clerks (ie, those in their fourth and fifth years of training) and (2) medical interns (ie, those in their sixth and seventh years of training). Under the supervision of clinical professors, medical interns were mentored by faculty staff. Medical interns also helped with supervising the medical clerks. All interviewers completed a 40-hour crash course. The educational topics in the course were tailored toward COVID-19–related prevention, care, and treatment. The topics included methods for taking a complete history, methods for conducting complete physical examinations, essential practices for COVID-19, guidance on precautionary measures for providing home care and isolating patients and other household members, and the assessment of high-risk conditions (ie, interviewers were given a referral guide for each medical condition). Data collection procedures were pilot-tested with patients through role play to ensure that data were collected consistently. During the phone calls, patients and their household members were educated on home care procedures and isolation guidelines. All clinical signs and symptoms were closely examined and recorded. Treatment regimens and procedures were adjusted accordingly, and patients were instructed to stay at home or return to the emergency room if they or their household members exhibited critical symptoms.

#### Statistical Analysis

Data entries were double-checked and cleaned by using STATA version 15 (StataCorp LLC). For the purposes of this prospective cohort study, descriptive statistics, including relative frequencies for categorical variables and means and standard deviations for quantitative variables, were reported. However, for the purposes of future studies that derive data from our cohort, associations will be examined by using appropriate regression analyses.

#### Ethics

The study protocol was reviewed and approved by the ethics committee of the Shahid Beheshti University of Medical Sciences (Ethics approval reference number: IR.SBMU.RETECH.REC.1399.114).

## Results

Of the 950 patients with confirmed COVID-19 who were approached at the initial phase of the Tele-COVID-19 study, 823 (86.6%) consented and were successfully enrolled in this study. Of the 823 participants, 261 (31.7%) were from Shohada Tajrish Hospital, 213 (25.9%) were from Loghman Hakim Hospital, 233 (28.3%) were from Ayatollah Taleghani Hospital, and 116 (14.1%) were from Shahid Modarres Hospital. The baseline characteristics of the enrolled patients are presented in Table 1.

Of the 823 participants, 449 (54.5%) were male. The mean age of participants was 50.1 years (SD 12.6 years). Overall, 65 (65/821, 7.9%) participants were health care workers, and 19 (19/821, 2.3%) participants reported that they travelled to known epicenters of COVID-19 within the previous 14 days of the interview. A total of 471 (471/818, 57.6%) reported that they were exposed to a patient with COVID-19 within the past 14 days of the interview. A total of 701 (701/814, 86.2%) patients visited a hospital due to suspicious signs and symptoms, and 59 (59/814, 7.2%) patients sought medical attention due to being exposed to people with probable COVID-19. Overall, 167 (167/811, 20.6%) were current/former smokers. Most patients did not have any underlying diseases (372/676, 55%), and only 25 (25/818, 3.1%) were immunodeficient. The mean length of hospitalization was 5.23 days (SD 4 days).

Detailed data on baseline clinical symptoms was available for 676 patients. Among the baseline clinical symptoms, the 3 most common symptoms were cough (466/676, 68.9%), respiratory distress (394/676, 58.3%), and fever (364/676, 53.8%). Only a small proportion of patients (29/676, 4.3%) had severe conditions (ie, admitted to an intensive care unit or had an oxygen saturation level of <90%). Most patients reported that they would be able to self-isolate at postdischarge (565/676, 83.5%).

**Table 1:** Baseline characteristics of patients with COVID-19 from the Tele-COVID-19 cohort in Tehran, Iran.

Characteristics		Value
**Number of patients from each hospital (N=823), n (%)^a^**		
	Loghman Hakim Hospital	213 (25.9)
	Shohada Tajrish Hospital	261 (31.7)
	Shahid Modarres Hospital	116 (14.1)
	Ayatollah Taleghani Hospital	233 (28.3)
**Sex (N=823), n (%)^a^**		
	Male	449 (54.6)
	Female	374 (45.4)
Age (years; N=823), mean (SD)		50.1 (12.6)
**Health care worker (N=821), n (%)^a^**		
	Yes	65 (7.9)
	No	756 (92.1)
**Travel history in the previous 14 days (N=821),n (%)^a^**		
	China	1 (0.1)
	Qom province	10 (1.2)
	Gilan province	8 (0.9)
	No travel history	802 (97.6)
**Exposure to patients with confirmed COVID-19 in the previous 14 days (N=818), n (%)^a^**		
	Yes	176 (21.5)
	No	642 (78.5)
**Reason for hospital visit (N=814), n (%)^a^**		
	Suspicious clinical signs and symptoms	701 (86.2)
	Exposure to a probable COVID-19 patient	59 (7.2)
	Other	54 (6.6)
**Smoking history (N=811), n (%)^a^**		
	Current smoker	77 (9.5)
	Former smoker	90 (11.1)
	Nonsmoker	644 (79.4)
**Chronic respiratory conditions^b^ (N=818), n (%)^a^**		
	Yes	81 (9.9)
	No	737 (90.1)
**Diabetes mellitus (N=820), n (%)^a^**		
	Yes	159 (19.4)
	No	661 (80.6)
**Cardiovascular conditions (N=817), n (%)^a^**		
	Yes	181 (22.2)
	No	636 (77.8)
**Chronic renal conditions (N=821), n (%)^a^**		
	Yes	57 (6.9)
	No	764 (93.1)
**Chronic liver conditions (N=821), n (%)^a^**		
	Yes	23 (2.8)
	No	798 (97.2)
**Immunodeficiency (N=818), n (%)^a^**		
	Yes	25 (3.1)
	No	793 (96.9)
**Underlying neurological conditions^c^ (N=819), n (%)^a^**		
	Yes	52 (6.5)
	No	767 (93.5)
Number of hospitalization days, mean (SD)		5.32 (4)
**Clinical signs and symptoms (N=676), n (%)^a^**		
	Fever	364 (53.8)
	Chills	327 (48.4)
	Myalgia	225 (33.3)
	Headache	306 (45.3)
	Cough	466 (68.9)
	Respiratory distress	394 (58.3)
	Nausea and vomiting	270 (39.9)
	Diarrhea	239 (35.4)
	Loss of appetite	289 (42.8)
	Loss of weight	69 (10.2)
	Abdominal pain	144 (21.3)
	Anosmia	127 (18.8)
	Ageusia	134 (19.8)
	Rhinorrhea	117 (7.9)
	Sore throat	171 (17.3)
	Consciousness alterations	95 (14.1)
**COVID-19 severity^d^ (N=676), n (%)^a^**		
	Mild to moderate	647 (95.7%)
	Severe	29 (4.3%)

^a^Percentages are rounded to 1 decimal point.

^b^Chronic respiratory conditions include asthma, emphysema, and chronic obstructive pulmonary disease.

^c^Underlying medical conditions include chronic neurological diseases and neurodevelopmental/intellectual disability.

^d^Severe cases included people who were admitted to the intensive care unit or had an oxygen saturation level of <90%.

During the initial data collection phase, more than 5000 phone calls were made. Overall, 577 reports were recorded in the daily critical case report sheets. Patients with critical conditions were directly followed by medical interns and clinical professors for more specific medical care. Patients with serious conditions (n=69) were referred to the emergency department, in accordance with hospital staff recommendations. Of these 69 patients, 40 (58%) were rehospitalized. Patients with minor conditions who primarily intended to revisit a hospital were successfully managed over the phone, leading to the prevention of unnecessary hospital visits (296/823, 36%). A total of 60 (60/823, 7.3%) patients who reported that they were experiencing adverse reactions to medications were managed through phone calls.

## Discussion

### Principal Findings

The Tele-COVID-19 cohort provided a platform for effectively following up with patients with COVID-19 after hospitalization. Our prospective cohort study presents a cost-effective way of managing postdischarge patients with COVID-19 and supporting them and their family members on their path to full recovery. The Tele-COVID-19 cohort was designed and run by a group of volunteer medical students, who successfully followed up with 823 postdischarge patients with COVID-19 during the baseline wave of this study. Given the burden of COVID-19 on health care systems in resource-limited settings such as Iran, telephone-based follow-up studies that involve medical students may not only enhance patient care, but also enhance medical education for medical students, who are often left out of the COVID-19 response due to concerns about limited personal protective equipment resources and students’ safety [15]. Early, postdischarge telephone follow-up calls have previously been shown to improve patients’ health outcomes and reduce their chances of readmission or critical condition development in the first month after discharge [16-19].

### Limitations

We acknowledge that our study has several limitations that are common among studies of a similar nature. First, our nonrandom sample of participants from major hospitals in Tehran may not be generalizable to patients with COVID-19 in other parts of Tehran or Iran. However, it is likely that the characteristics of the participants who were recruited into our cohort are not considerably different from those of patients from other Tehran hospitals that were not included in our study. Second, participants' self-reported responses with regard to potential risk factors or underlying comorbidities are prone to social desirability and reporting biases. Third, we did not collect any data on the mental health profiles of patients. This could be considered for future waves of data collection. Fourth, most patients in our cohort (647/676, 95.7%) exhibited mild or moderate COVID-19 symptoms. Furthermore, these patients had no adverse underlying conditions. Therefore, these patients do not provide a complete clinical picture of severe COVID-19 in Tehran hospitals.

### Conclusions

The Tele-COVID-19 cohort is a unique, student-led cohort that could provide an effective platform for improving our evolving understanding of COVID-19 care and treatment in Iran. Such cohort studies assist the medical community by reducing the number of medical complications among people who are recovering from COVID-19, improving our understanding of the clinical course of the disease, identifying potential drug interactions and the adverse effects of pharmacotherapies, reducing the household transmission and secondary attack rates of SARS-CoV-2, referring discharged patients with critical conditions to the emergency department in a timely manner, reducing patients’ anxiety, and preventing unnecessary hospital visits. Moreover, cohorts like the Tele-COVID-19 cohort provide a cost-effective and rapidly implementable platform for improving our understanding of COVID-19 in resource-limited settings.
